# Gaussian Quadrature Formulae for Arbitrary Positive Measures

**Published:** 2007-02-15

**Authors:** Andrew D. Fernandes, William R. Atchley

**Affiliations:** 1 Graduate Program in Biomathematics; 2 Center for Computational Biology; 3 Department of Genetics, North Carolina State University Raleigh, NC 27695-7614

## Abstract

We present computational methods and subroutines to compute Gaussian quadrature integration formulas for arbitrary positive measures. For expensive integrands that can be factored into well-known forms, Gaussian quadrature schemes allow for efficient evaluation of high-accuracy and -precision numerical integrals, especially compared to general *ad hoc* schemes. In addition, for certain well-known density measures (the normal, gamma, log-normal, Student’s *t*, inverse-gamma, beta, and Fisher’s *F*) we present exact formulae for computing the respective quadrature scheme.

## Motivation

This paper is concerned with the efficient and accurate calculation of likelihood integrals of the form

(1) $$\text{Pr}\;(H|D)\propto \int_{h\in H}\text{Pr}(D|h)\cdot \text{Pr}\;(h)dh,$$

through the construction of a Gaussian-type quadrature scheme that is optimized specifically for the known prior distribution Pr(*h*). Our specific motivation stems from studies in the molecular evolution of protein sequences where it is important to take variation of evolutionary rates among sites into account when inferring phylogenies. In the context of this specific problem, both Felsenstein (2001; Felsenstein 2004) and Mayrose et al. (2005) pointed out that Gaussian quadrature formulae can be used to provide more accurate and more rapidly convergent numerical integration methods than the more common “equal percentile” method of Yang (1994). Unfortunately, Gaussian-type quadrature formulae have only been derived for a relatively small number of prior distributions. In the context of molecular evolution, the two most common priors are the gamma and log-normal distributions. Gaussian quadrature formulae for the gamma distribution are already known as “Generalized Gauss-Laguerre” quadrature (Felsenstein, 2001), although admittedly the mathematical similarity between these schemes is not obvious with the usual formulation of Gauss-Laguerre quadrature. Thus their equivalence is generally not appreciated. Unfortunately, until now explicit Gaussian quadrature formulae were not available for log-normal (or other) priors commonly used in computational biology.

The purpose of this paper is to provide an efficient and rapid algorithm with accompanying computer library that permits computation of Gaussian quadrature rules for *arbitrary* prior distributions. In some cases, we derive analytic formulae for specific common distributions. Although motivated by a specific application to integrals found in the field of molecular evolution, we stress that our methods (and computer code) are applicable to the solution of numerical integration problems in general.

## Problem Statement

We wish to find a set *i* = 0, 1, 2, …, (*n* − 1) of weights *w_i_* and abscissae *x_i_* such that the approximation

(2) $$\int_{a}^{b}w(x)\cdot f(x)dx\approx \sum_{i=0}^{n-1}w_{i}\cdot f(x_{i})$$

is exact whenever *f* is a polynomial of degree 2*n* − 1 or less, and *w*(*x*) is a known “weight function.” In our case *w*(*x*) represents the positive density measure of our prior likelihood. A good and complete modern reference covering the theory of Gaussian (and related) types of quadrature rules can be found in Gautschi (2004). If *f* is expanded as a polynomial series, inspection suggests that any quadrature scheme will depend on the raw moments of *w*(*x*). Indeed, defining the (real) inner product

(3) 〈f|g〉=∫f(x)g (x)·w (x) dx,

it is well known that there always exists a set of polynomials, orthogonal with respect to this inner product, such that

(4) $$\begin{array}{c} p_{-1}=0,\;\;\;p_{0}=1\\ p_{i+1}(x)=(x-a_{i})\cdot p_{i}(x)-b_{i}\cdot p_{i-1}(x),\;\;\;i=0,1,2,\ldots \end{array}$$

and where the recurrence coefficients *a_i_* and *b_i_* can be calculated explicitly from

(5) $$\begin{array}{l} a_{i}=\frac{\langle x\cdot p_{i}|p_{i}\rangle }{\langle p_{i}|p_{i}\rangle },\;\;\;i=0,1,2,\ldots \\ b_{i}=\frac{\langle p_{i}|p_{i}\rangle }{\langle p_{i-1}|p_{i-1}\rangle },\;\;\;i=1,2,\ldots \end{array}$$

with the coefficient *b*_0_ being arbitrary and set by convention such that *b*_0_ = ∫*w*(*x*) *dx.* Therefore the first *n* recursion coefficient pairs are uniquely determined by the first 2*n* moments of the measure *w*. Once the coefficients *a_i_* and *b_i_* are known, they can be assembled into the tridiagonal Jacobi matrix

(6) $$J=\left[\begin{array}{cccccc} a_{0} & \sqrt{b_{1}} & & & & \\ \sqrt{b_{1}} & a_{1} & \sqrt{b_{2}} & & & \\ & \sqrt{b_{2}} & \ddots & \ddots & & \\ & & \ddots & \ddots & \sqrt{b_{n-2}} & \\ & & & \sqrt{b_{n-2}} & a_{n-2} & \sqrt{b_{n-1}}\\ & & & & \sqrt{b_{n-1}} & a_{n-1} \end{array}\right].$$

The desired abscissae *x_i_* are then equal to the eigenvalues of *J*, and the desired weights are given by the relationship

(7) $$w_{i}=b_{0}\cdot q_{i,0}^{2},$$

where *q_i,_*_0_ is the first component of the normalized eigenvector *q_i_* of matrix *J*.

## Solution Methods

Formulae are known that explicitly express the recursion coefficients *a_j_* and *b_j_* in terms of the raw moments of *w*. Unfortunately, these explicit representations are extremely ill-conditioned and thus are not usable even for “well behaved” weight functions or quadrature schemes of fairly low order *n*. If the integrals of Equation (5) can be calculated efficiently and accurately, Stieltjes’ Procedure calculates the recursion coefficients via iterative application of Equations (4) and (5) forming the sequence {*p*_−1_, *p*_0_}→{*a*_0_, *b*_1_}→{*p*_1_}→{*a*_1_, *b*_2_}→{*p*_2_}→…. Athough better behaved than explicit computation, Stieltjes’ Procedure also tends to be moderately ill-conditioned (Press, Teukolsky et al. 1997) and therefore of limited value. Alternatively, the Sack-Donovan-Wheeler algorithm (Press, Teukolsky et al. 1997) has been suggested as a way to overcome the numerical instabilities inherent in Stieltjes’ Procedure by utilizing modified moments rather than raw moments in Equations (4) and (5). The downside of the Sack-Donovan-Wheeler algorithm is its reliance on the *a priori* selection of a “good” polynomial basis for the moments of *w*(*x*), in itself a fairly difficult and subjective procedure that is dependent on heuristic approximation of the moments of *w*(*x*). Forming such an approximation may be as or more difficulty than solving the original problem.

Recently, a general-purpose and unconditionally stable algorithm to calculate Gaussian weights and abscissae for any positive measure has been proposed (Gander and Karp, 2001). The method is based on the observation (Boley and Golub, 1987) that the *discrete* measure

(8) $$\omega_{m}(x)=\sum_{i=0}^{m-1}\omega_{i}\cdot \delta (x-\xi_{i})$$

can have its weights and abscissae assembled into a sparse matrix

(9) $$W_{m}=\left[\begin{array}{cccccc} 1 & \sqrt{\omega_{0}} & \sqrt{\omega_{1}} & \cdots & \sqrt{\omega_{m-2}} & \sqrt{\omega_{m-1}}\\ \sqrt{\omega_{0}} & \xi_{1} & & & & \\ \sqrt{\omega_{1}} & & \xi_{2} & & & \\ \vdots & & & \ddots & & \\ \sqrt{\omega_{m-2}} & & & & \xi_{m-2} & \\ \sqrt{\omega_{m-1}} & & & & & \xi_{m-1} \end{array}\right]$$

that is orthogonally similar to the Jacobi matrix

(10) $$J_{m}=\left[\begin{array}{ccccccc} 1 & \sqrt{b_{0}} & & & & & \\ \sqrt{b_{0}} & a_{0} & \sqrt{b_{1}} & & & & \\ & \sqrt{b_{1}} & a_{1} & \sqrt{b_{2}} & & & \\ & & \sqrt{b_{2}} & \ddots & \ddots & & \\ & & & \ddots & \ddots & \sqrt{b_{m-2}} & \\ & & & & \sqrt{b_{m-2}} & a_{m-2} & \sqrt{b_{m-1}}\\ & & & & & \sqrt{b_{m-1}} & a_{m-1} \end{array}\right],$$

where *b*_0_ is the *ω_m_*-measure of the entire domain of *ω_m_*. Gander and Karp showed that if a sequence of discrete measures given by Equation (8) converged to a given continuous measure such that $\underset{m\to \infty }{\text{lim}}\omega_{m}(x)=w(x)$, then *J_m_* would similarly converge to the recurrence coefficients of the continuous measure. Such convergence had already been noted and exploited several years before in the OrthPol software package (Gautschi, 1994). A re-implementation, modernization, and modification of some of Gautschi’s algorithms form the core of our work. To continue, given *J_m_*, standard eigen-decomposition algorithms for symmetric tridiagonal matrices can be used to compute the Gaussian quadrature weights and abscissae for the given weight function. In summary, the weights and abscissae of an arbitrary positive measure *w*(*x*) can be as determined by first finding a discrete *ω_m_*(*x*) tht approximates *w*(*x*) “well enough”, using the Lanczos reduction algorithm to transform *W_m_* → *J_m_*, concomitantly obtaining the recursion coefficients {*a_i_,b_i_*}, and then eigen-decomposing *J_m_* to determine the final weights and abscissae {*x_i_,w_i_*}via Equation (7).

## Algorithmic Details

The implementation details for the overall process, starting from a given weight function and ending with a set of Gaussian quadrature weights and abscissae, are best elucidated by a worked example. Assume we are given the weight function *w*(*x*) ∝ *e*^−^*^x^*, *x* ≥ 0, where we do not know the normalization constant 1/∫*w*(*x*)*dx* and do not recognize *e*^−^*^x^* as the weight function for the well-known Gauss-Laguerre quadrature scheme. Our first step is to select a sequence of measures, as per Equation (8), that converges to the measure *e*^−^*^x^* *dx*. Following Gautschi (1994), we use a classical numerical integration scheme to approximate ∫*w* (*x*)*dx*, namely the Fejér Type-2 integration rule (Gautschi originally used the Fejér Type-1 rule). Fejér integration rules are very similar to the well-known Clenshaw-Curtis integration rules over the domain *z* ∈ [−1,1]. However, the Fejér rules are open-ended, do not require evaluation at the domain endpoints, and are therefore more suitable for measures with non-compact support. Fejér Type-2 rules also have an efficiency advantage over the Type-1 rules in the fact that the *n*-point Type-2 abscissae are an interleaved subset the (2*n* +1)-point Type-2 abscissae. Therefore, the Type-2 rules allow us to reuse all previously calculated ordinates when the number of integration points is doubled. Lastly, Fejér Type-2 integration weights can be calculated very rapidly via real inverse Fast Fourier Transform (Waldvogel 2006), allowing a large number of points to be efficiently utilized in approximating ∫*w* (*x*)*dx*. The supplied subroutine fejer2_abscissae calculates the required abscissae and integration weights {*z_i_*, *q_i_*} for a given number of abscissae *i* = 0, 1, 2, …, (*m* − 1). The transformation $g\;(z)=\frac{\left(1+z\right)}{\left(1-z\right)}$ is used via the subroutine map_fejer2_domain to map *z* ∈ (−1,1) → *x* ∈ (0,∞) and change the variable of integration such that ∫_0_^∞^*e*^−^*^x^dx*=∫_−1_^+1^*e*^−^*^g^*^(^*^z^*^)^*g*′(*z*)*dz*, giving the final abscissae and weights {*ξ_i_*, *ω_i_*}for Equation (9), where *ξ_i_* = *g*(*z_i_*) and *ω_i_* = *q_i_* · *w*(*g*(*zi*)). *g*′ (*z_i_*). Note that the subroutine map_fejer2_domain is capable of mapping the Fejér interval to other arbitrary finite and non-finite domain intervals in addition to the particular transformation *g*(*z*) utilized here.

The tridiagonalization of *W_m_* in Equation (9) to *J_m_* in Equation (10) can be accomplished by using the subroutine lanczos_tridiagonalize, a subroutine that exploits the sparsity structure of Equation (9) via the Lanczos algorithm (Golub and Van Loan, 1996) for efficient tridiagonalization. Lastly, the eigen-decomposition of *J_m_* in Equation (10) and subsequent calculation of the final Gaussian quadrature rule for *w*(*x*) via Equation (7) is accomplished by use of the subroutine gaussqr_ from_rcoeffs, where the eigen-decomposition is performed using a modified implicit-shift QL algorithm. Note that the coefficient *b*_0_ returned from lanczos_tridiagonalize estimates ∫*w*(*x*) *dx* for the given *m*. Thus, we can set *b*_0_ = 1 prior to calling gaussqr_from_rcoeffs to normalize *w*(*x*) wihout explicitly knowing or calculating the actual normalization coefficient. In many cases, this can significantly speed up the calculation of *w*(*x*). For common distributions such as the normal, gamma, log-normal, and others, the utility function standard_distribution_rcoeffs is supplied to compute recursion coefficients directly.

Lastly, we must ensure that *m* is large enough so that *ω_m_* (*x*) aproximates *w*(*x*) sufficiently closely to further ensure that the *i* = 0, 1, 2, … (*n* − 1) < *m* computed quadrature points{*x_i_,w_i_*} coverge. The subroutine relative_error computes the maximum relative error between its two vector arguments. Since *w_i_* is guaranteed to be positive for all non-negative measures *w*(*x*), it suffices (and simplifies matters) to verify convergence of *w_i_* wihout explicit regard to the convergence of *x_i_*.

## Implementation Details

In using the subroutines presented, there are a few subtleties in the overall procedure that can be exploited in order to address non-standard situations or increase computational efficiency. First, we note that the discrete measure denoted by Equation (8) can be used to approximate *any* finite union of disjoint intervals. For instance, if we wished to use the (admittedly contrived) implicit weight function

(11) $$w(x)\propto \{\begin{array}{cc} e^{-x}, & 0\leq x<1\\ 1/x^{2}, & 1\leq x \end{array}$$

over support 0 ≤ *x*. Or subroutines could be applied twice, once for each continuous interval, yielding two discrete-measure approximations, each with approximate normalization consonant. The two discrete measures could then be combined into a set of abscissae and weights {*ξ_i_*, *ω_i_*} tht would then be subject to the Lanczos tridiagonalization procedure in order to determine the recursion coefficients of Equation (11). Note that the normalization of Equation (11) is computed “on the fly” and therefore allows great flexibility in choosing the weight function *w*(*x*). Furthermore, note that the example weight function of Equation (11) is not even continuous at *x* = 1.

Second, we note that computing an *m*-node Fejér Type-2 integration scheme is done by performing a real inverse fast Fourier transform of size (*m* + 1). Athough the subroutine supplied is capable of computing inverse Fourier transforms of almost arbitrary size, the transform is efficient *only* if (*m* + 1) has divisors from the set {2, 3, 4, 5}. To further increase efficiency, we note that the Fejér Type-2 nodes are simple to compute via

(12) $$z_{i}=\text{cos}\left(\frac{(i+1)\cdot \pi }{m+1}\right),\;\;\;i=0,1,\ldots ,(m-1),$$

implying that an *m_1_* -point and *m*_2_ -point integration scheme will share common abscissae if (*m*_1_ +1) and (*m*_2_ + 1) have a common divisor. Having common abscissae imply that previously computed values of *w*(*g*(*z_i_*)) could be reused as *m* increases, thus increasing the efficiency of approximating *w*(*x*). Therefore the recommended sequence of *m* for fejer2_abscissaefollows {3,7,15,31,63, …}. For very simple, well-behaved weight functions, it may be preferable to simply use *m* of a few hundred or few thousand, and not worry excessively about convergence when *m* is small. Such an approach may be indicated when pre-computing quadrature schemes for a parameterized family of weight functions; the shape parameter of the unit-mean gamma distribution, for example. Rather than determining quadrature points for every desired shape parameter, it may make more sense to pre-compute weights and abscissae as functions of the shape parameter at particular parameter values, and then interpolate a quadrature scheme for all “in-between” parameter values. Obviously, Fejér nodes and weights can be pre-computed as well.

There may be situations where it is useful to know the analytic form of a particular weight function’s recursion coefficients. In particular, well-known density functions can often have their recurrence relationships determined by Stieltjes’ Procedure, and a representative sample of such is shown in Table 1. Recursion coefficients computed from this table can be supplied directly to subroutine gaussqr_from_rcoeffs, although better numeric stability may be achieved by approximating these densities via standard_distribution_rcoeffs. Note that Gaussian quadrature schemes may not exist for all distributions at all parameter values. In these cases, non-existence of the quadrature scheme is due to the non-existence of the distribution’s relevant higher-order moments. In any case, caution should be exercised in utilizing Table 1 for these distributions lest numerical truncation error inadvertently become too great. Lastly, as Table 1 shows, it is often possible to extract a common factor *λ* from the recursion coefficients. Such a common factor merely scales the eigenvalues of *J_m_* while leaving the eigenvectors alone, and thus may be safely ignored prior to eigen-decomposition.

We conclude with a reminder that our choice of the Fejér Type-2 integration points for computing the approximation $\underset{m\to \infty }{\text{lim}}\omega_{m}(x)=w(x)$ is quite arbitrary, and other integration schemes may be more appropriate given a different family of weight functions. For instance, a simple 1/*m* “equal-percentile” approach, reminiscent of Yang (1994), may be more efficient than a Fejér-like scheme for weight functions with numerous sharp peaks. Further, rational-quadrature schemes may be a better choice for measures with poles near the measure’s support (Gautschi, 1999; Weideman and Laurie, 2000; Van Deun, Bultheel et al. 2006). In any case, the Fejér Type-2 scheme utilized here should prove adequate for most common weight functions utilized in likelihood calculations today.

## Usage Guidelines

Two approximations must be made to construct a set of quadrature abscissae and weights. First, the number of discrete points that will be used to approximate the weight function must be chosen. Second, the number of quadrature points to compute the final likelihood integral must be chosen. In this section, we provide guidance on how to select the appropriate number of points in each case.

First, when approximating *w*(*x*) by a discrete measure, we exploit efficiencies inherent in the FFT and sparsity structure of matrices *W_m_* and *J_m_* to quickly and efficiently approximate *w*(*x*) wih thousands (1023, 2047, or more) points. For example, using 1023 points to approximate a standard *N*(0, 1) distribution results in quadrature coefficients, correct to within one part in 2×10^−15^ (the limit of machine precision), to be calculated in negligible time compared to all but the most trivial phylogenetic likelihood calculations.

Guidance for the second case, the number of quadrature points to use, is more difficult to give because of the main convergence property of Gaussian quadrature: the rate of convergence depends critically on how well the integrand can be approximated by a polynomial. The better the approximation, the more rapid the convergence. Unfortunately, the converse is also true; functions that are poorly approximated by polynomials may have far *worse* convergence characteristics than other numerical integration schemes. The best guidance on picking the number of quadrature points for a particular integrand may come from trial and error: keep increasing the number of points until numerical convergence seems to be achieved. This empirical “try it and see” approach has been utilized by Yang (1994), Mayrose et al. (2004), among others and is commonly advised.

In an effort to provide a more concrete example of how Gaussian quadrature fares in a sample integrand from molecular evolution studies, consider one site of a four sequence alignment where every nucleotide is different (one each of A, C, G, and T), and we know *a priori* that all four sequences share an unknown common ancestor one time unit in the past. Assuming a normalized Jukes-Cantor (1969) model of evolution yields a likelihood function of

(13) $$f(r)\propto (1+3\cdot e^{-\frac{4}{3}r}){\left(1-e^{-\frac{4}{3}r}\right)}^{3}$$

for a given evolutionary rate *r*. We assume unit-proportionality for convenience. Further assuming that rates are distributed according to a unit-mean Gamma distribution with coefficient of variation $\sqrt{2}$ results in a weight function of

(14) $$w(r)=4\cdot r\cdot e^{-2r}.$$

The likelihood of our data given our model can then be calculated analytically, resulting in

(15) $$\int_{0}^{\infty }h(r)dr=\frac{30080}{53361}\approx 0.5637076,$$

where

(16) h(r)=w(r)·f(r).

**Figure 1:**
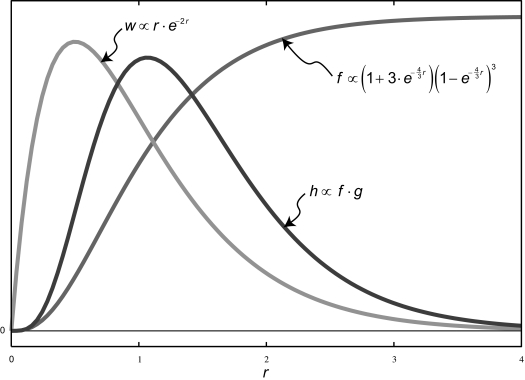
A graphical depiction of the relative shapes of Equations (13), (14), and (16).

**Figure 2:**
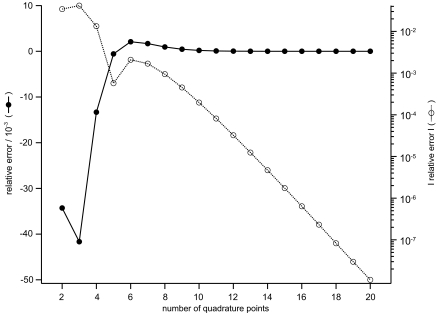
The number of quadrature points versus the relative error in the sample molecular evolution integration problem.

A graph depicting the relative shapes of *f*, *g*, and *h* is shown in Figure 1. A plot of the relationship between the number of quadrature points and the relative error of the integral in Equation (15) is shown in Figure 2. Seven quadrature points result in a relative error of about 0.15 %, and twenty points result in a relative error of about 1.1 × 10^−6^ %. Note that seven or more quadrature points demarks the asymptotic domain for numerical convergence where the error decreases polynomially with the number of quadrature points.

**Figure 3:**
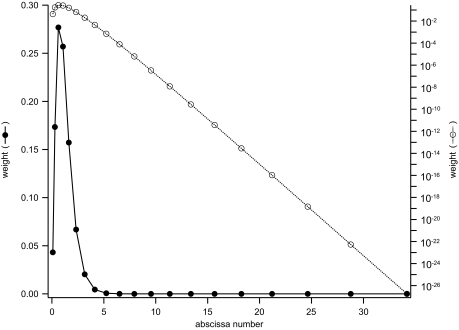
Gaussian quadrature weights and abscissae *n* = 20 for points for the sample molecular evolution integration problem.

A detailed examination of the twenty-quadrature point case shows an interesting optimization that applies to likelihood functions such as Equation (13), where the likelihood approaches a constant value as its argument approaches infinity. Recall that Gaussian quadrature schemes are designed to optimally integrate polynomials *p*(*x*), and that complex analysis tells us that for polynomials, |*p*(*x*)| → ∞ as |*x*|→∞. For *w*(*x*) · *p*(*x*) to be integrable, |*w* (*x*)|→0 relatively rapidly as |*x*|→∞. Therefore we expect the quadrature weight *w_i_* to rapidly become very small as the magnitude of its respective abscissa *x_i_* inreases. An illustration of the magnitudes of {*x_i_*, *w_i_*} for a twenty-point quadrature scheme for our *h*(*r*) example, above, is shown in Figure 3. Note that after the first ten to twelve abscissae have been summed, the contribution of the remaining eight to ten points will be negligible; the integration scheme assumes that *f*(*r*) will be polynomially large when in fact it is almost constant. Thus we can gain the accuracy benefits of using a twenty-point integrator while incurring the cost of only ten evaluations of *f*(*r*).

## Figures and Tables

**Table 1:**
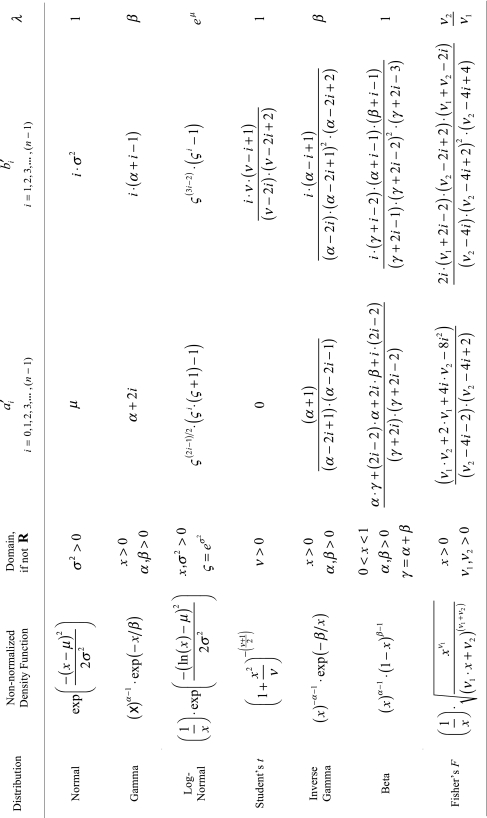
Exact recursion coefficients for selected probability distributions. For Equations (6) and (7), we scale the recursion coefficients such that *a_i_*= *λ* · *a*′*_i_* and *b_i_* = *λ*^2^ · *b*′*_i_*. Note that for *n* recursion coefficients, at least the first 2*n* moments must exist. There is also a special case for the Beta distribution:*a_0_* = 1/2 when *α* = *β* = 1 (the uniform distribution).
